# Short-term psychological support to civilians exposed to the January 2015 terrorist attacks in France

**DOI:** 10.1192/j.eurpsy.2022.274

**Published:** 2022-09-01

**Authors:** C. Vuillermoz, P. Pirard, L. Stene, S. Vandentorren

**Affiliations:** 1 Sorbonne Université, Inserm, Institut Pierre Louis D’épidémiologie Et De Santé Publique, Iplesp, Social Epidemiology Research Team, Paris, France; 2 Santé publique France, Direction Des Maladies Non Transmissibles Et Traumatismes, Saint Maurice, France; 3 Norwegian Centre for Violence and Traumatic Stress Studies, Section For Trauma, Catastrophes And Forced Migration - Children And Youths, Oslo, Norway; 4 Santé publique France, Direction Scientifique Et Internationale, Saint Maurice, France

**Keywords:** Psychological support, Epidemiology, mental health, Terrorist attack

## Abstract

**Introduction:**

Some projects have described post-disaster psychosocial services and planning across Europe. However, little is known about the real psychosocial disaster responses such as low-intensity initiatives after a terrorist attack

**Objectives:**

This study aims (1) to describe psychological support (PS) in the immediate (<48 hours), post-immediate (48 hours – 1 week) periods and more than one week after a terrorist attack among terror-exposed people, and (2) to identify factors associated with a lack of short-term PS among those who suffered from mental health disorders.

**Methods:**

This study used data from a longitudinal survey of 189 civilians exposed to the January 2015 terrorist attacks conducted 6 months after the attacks. Factors associated with lack of PS after the attacks was identified using a Robust Poisson regression in three separate models (for the 3 periods).

**Results:**

Among participants who suffered from PTSD (n=34), depression (n=74), or anxiety (n=59) 6-9 months after the terrorist attacks, respectively, 9%, 18% and 12% did not received psychological support. The lack of immediate PS was associated with geographical distance, type of exposure, and support in daily life. The lack of post-immediate PS was associated with geographical distance, peri-traumatic reactions and past psychological follow-up. The lack of PS after one week was associated with geographical distance and social isolation.

**Conclusions:**

Characteristics of exposition and social support seem to play an important role in lack of PS after a terrorist attack and highlights the need to use strategies to reach out to people regardless of the type of exposure.

**Disclosure:**

No significant relationships.

